# Assessing the Capability of ChatGPT in Answering First- and Second-Order Knowledge Questions on Microbiology as per Competency-Based Medical Education Curriculum

**DOI:** 10.7759/cureus.36034

**Published:** 2023-03-12

**Authors:** Dipmala Das, Nikhil Kumar, Langamba Angom Longjam, Ranwir Sinha, Asitava Deb Roy, Himel Mondal, Pratima Gupta

**Affiliations:** 1 Microbiology, All India Institute of Medical Sciences, Deoghar, Deoghar, IND; 2 Pathology, All India Institute of Medical Sciences, Deoghar, Deoghar, IND; 3 Microbiology, IQ City Medical College Hospital, Durgapur, IND; 4 Physiology, All India Institute of Medical Sciences, Deoghar, Deoghar, IND

**Keywords:** medical education, question, artificial intelligence, automated question-answering, competency-based medical education, second-order questions, first-order questions, microbiology, language model, chatgpt

## Abstract

Background and objective

ChatGPT is an artificial intelligence (AI) language model that has been trained to process and respond to questions across a wide range of topics. It is also capable of solving problems in medical educational topics. However, the capability of ChatGPT to accurately answer first- and second-order knowledge questions in the field of microbiology has not been explored so far. Hence, in this study, we aimed to analyze the capability of ChatGPT in answering first- and second-order questions on the subject of microbiology.

Materials and methods

Based on the competency-based medical education (CBME) curriculum of the subject of microbiology, we prepared a set of first-order and second-order questions. For the total of eight modules in the CBME curriculum for microbiology, we prepared six first-order and six second-order knowledge questions according to the National Medical Commission-recommended CBME curriculum, amounting to a total of (8 x 12) 96 questions. The questions were checked for content validity by three expert microbiologists. These questions were used to converse with ChatGPT by a single user and responses were recorded for further analysis. The answers were scored by three microbiologists on a rating scale of 0-5. The average of three scores was taken as the final score for analysis. As the data were not normally distributed, we used a non-parametric statistical test. The overall scores were tested by a one-sample median test with hypothetical values of 4 and 5. The scores of answers to first-order and second-order questions were compared by the Mann-Whitney U test. Module-wise responses were tested by the Kruskall-Wallis test followed by the post hoc test for pairwise comparisons.

Results

The overall score of 96 answers was 4.04 ±0.37 (median: 4.17, Q1-Q3: 3.88-4.33) with the mean score of answers to first-order knowledge questions being 4.07 ±0.32 (median: 4.17, Q1-Q3: 4-4.33) and that of answers to second-order knowledge questions being 3.99 ±0.43 (median: 4, Q1-Q3: 3.67-4.33) (Mann-Whitney p=0.4). The score was significantly below the score of 5 (one-sample median test p<0.0001) and similar to 4 (one-sample median test p=0.09). Overall, there was a variation in median scores obtained in eight categories of topics in microbiology, indicating inconsistent performance in different topics.

Conclusion

The results of the study indicate that ChatGPT is capable of answering both first- and second-order knowledge questions related to the subject of microbiology. The model achieved an accuracy of approximately 80% and there was no difference between the model's capability of answering first-order questions and second-order knowledge questions. The findings of this study suggest that ChatGPT has the potential to be an effective tool for automated question-answering in the field of microbiology. However, continued improvement in the training and development of language models is necessary to enhance their performance and make them suitable for academic use.

## Introduction

In recent years, natural language processing (NLP) and machine learning (ML) techniques have revolutionized the field of question-answering systems [1]. ChatGPT is one such artificial intelligence (AI) language model that has been trained to understand the nuances of natural language and provide accurate answers to a wide range of questions [2,3,4]. While ChatGPT has shown promising results in solving problems in various fields, its ability to accurately answer first- and second-order knowledge questions in microbiology has not been extensively evaluated. First-order questions are straightforward questions that ask for factual information or seek a direct answer. Second-order questions, on the other hand, are more complex and require higher-level thinking and interpretation. These questions demand analysis or evaluation of information or an opinion or prediction based on evidence [5].

Microbiology is a fundamental subject in medical education and it deals with the study of microorganisms, including bacteria, viruses, fungi, and parasites. Medical students need to have a sound understanding of microbiology as it is directly related to the diagnosis and treatment of infectious diseases [6]. Competency-based medical education (CBME) is an approach to medical education that focuses on the acquisition of specific competencies rather than the general accumulation of knowledge. The CBME curriculum for microbiology includes a total of eight modules that aim to assess the student's understanding of the subject [7].

Automated question-answering systems have the potential to transform medical education by providing instant and accurate responses to complex questions [8]. The use of ChatGPT as an automated question-answering system in the field of microbiology could aid in the development of effective educational tools that can help medical students learn and understand the subject better. However, before implementing ChatGPT as an educational tool, it is crucial to evaluate its ability to accurately answer first- and second-order knowledge questions related to microbiology based on the CBME curriculum.

In light of this, in this study, we aimed to evaluate the performance of ChatGPT in answering first- and second-order knowledge questions related to microbiology. This could aid in the development of effective educational tools that can help medical students master the subject of microbiology. The results of this study could also shed light on the strengths and weaknesses of ChatGPT as an automated question-answering system with regard to the field of microbiology and pave the way for further research in this field.

## Materials and methods

Study design and setting

This cross-sectional observational study was conducted by the department of Microbiology, Pathology, and Physiology, All India Institute of Medical Sciences, Deoghar, Jharkhand, India in February 2023. We used an online AI language model - ChatGPT - to converse with and the responses were collected for analysis. The program is free for registered users at this point in time.

Ethical consideration

This study does not involve any human or animal research participants. Responses generated by the language model were not shared in this paper. Hence, according to current guidelines in the country, this study does not require any ethical clearance.

Question preparation

According to the CBME curriculum for the subject of microbiology, we prepared a set of first- and second-order questions. The researchers prepared a total of 96 questions, with six first-order and six second-order knowledge questions for each of the eight modules, as shown in Table 1 [9]. All questions were reviewed for content validity by an expert microbiologist.

**Table 1:** Modules of microbiology as per competency-based medical education CVS: cardiovascular system; MI: microbiology competency category

Number	Competency module
MI 1	General microbiology and immunity
MI 2	CVS and blood
MI 3	Gastrointestinal and hepatobiliary system
MI 4	Musculoskeletal system skin and soft tissue infections
MI 5	Central nervous system infections
MI 6	Respiratory tract infections
MI 7	Genitourinary and sexually transmitted infections
MI 8	Zoonotic diseases and miscellaneous

Data collection

The questions were used to converse with ChatGPT by a single user. The responses were recorded for further analysis. We did not use the “regenerate response” and took the first response to be the final answer.

Scoring of answers

**Figure 1:**
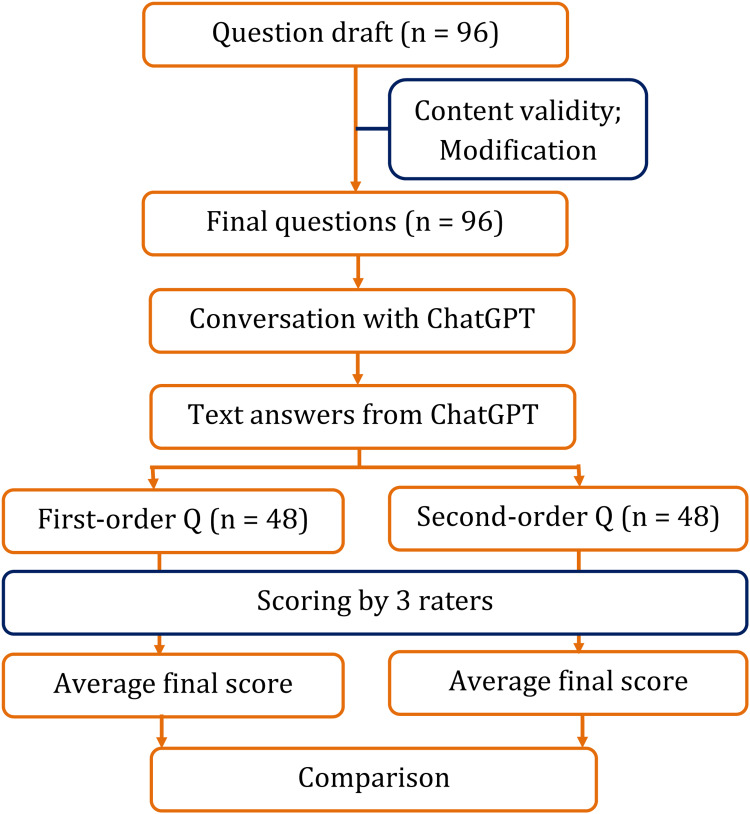
Brief study protocol from drafting questions to statistical analysis Q: Question; n: number

The responses generated by ChatGPT were scored by three microbiologists. The scoring was done on a rating scale of 0-5, where 0 indicates an incorrect answer and 5 indicates a fully correct answer. The average of the three scores was taken as the final score for each response. The brief study protocol is depicted in Figure 1.

Having multiple evaluators to assess an answer can help reduce biases and increase the accuracy and reliability of the evaluation. Different evaluators may have different biases based on their personal experiences, perspectives, or preferences. Having multiple evaluators can help mitigate these biases. Hence, we employed three evaluators to reduce biases.

Statistical analysis

As the data were not normally distributed, non-parametric statistical tests were used for analysis. The overall scores were tested using a one-sample median test with hypothetical values of 4 and 5. The scores of the first-order and second-order knowledge questions were compared using the Mann-Whitney U test. The module-wise responses were tested using the Kruskal-Wallis test, followed by the post hoc test for pairwise comparisons [10]. All the tests were conducted in GraphPad Prism 7 (Dotmatics, Boston, MA). A p-value <0.05 was considered statistically significant.

## Results

**Figure 2:**
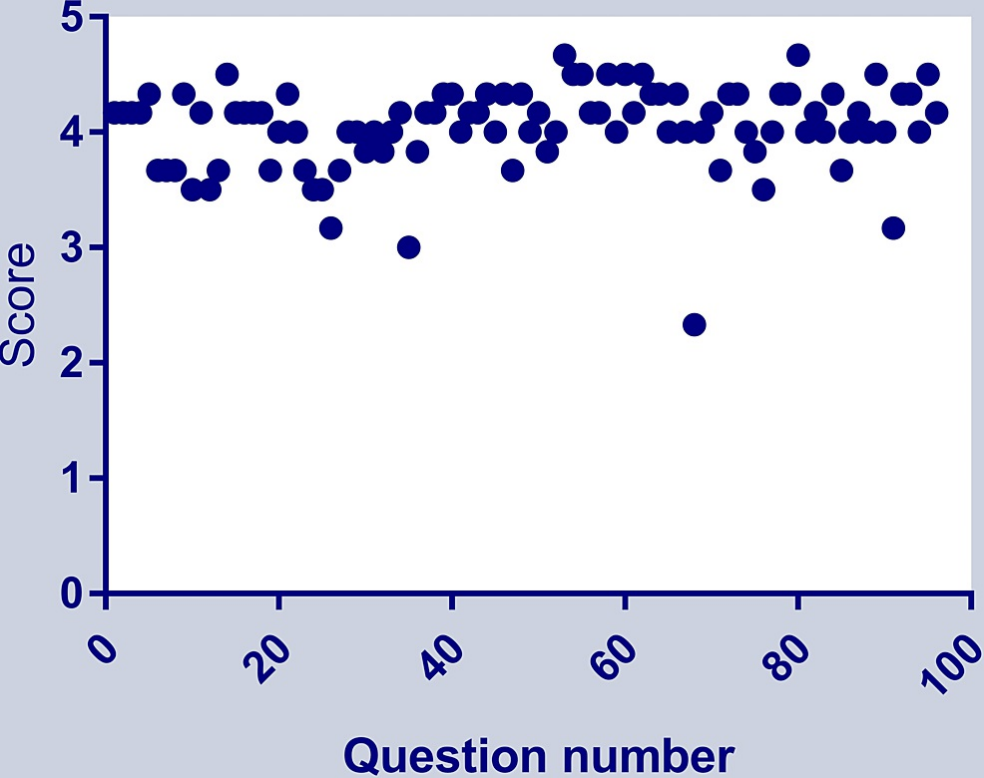
Frequency distribution of scores of 96 answers to both first- and second-order questions

The overall mean score of 96 answers was 4.04 ±0.37 (median: 4.17, Q1-Q3: 3.88-4.33). The frequency distribution of scores of 96 answers to both first-order and second-order knowledge questions is shown in Figure 2.

**Figure 3:**
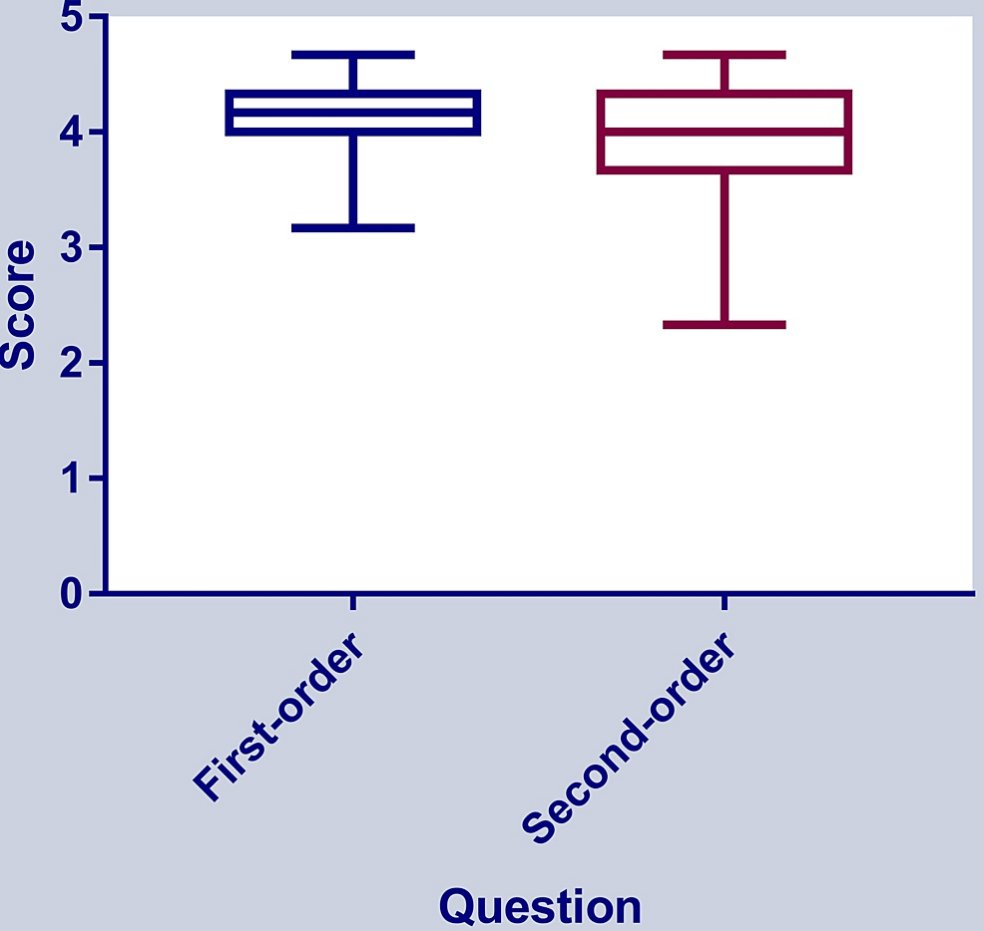
Comparison between scores of answers to first- and second-order knowledge questions

The mean score of the answers to first-order knowledge questions was 4.07 ±0.32 (median: 4.17, Q1-Q3: 4-4.33), and that of answers to second-order questions was 3.99 ±0.43 (median: 4, Q1-Q3: 3.67-4.33). The scores for answers to first- and second-order knowledge questions were similar (Mann-Whitney p=0.4), as shown in Figure 3.

The score was significantly below the score of 5 (one-sample median test p<0.0001) and similar to 4 (one-sample median test p=0.09), indicating an accuracy rate of 80%.

The highest score was obtained in the central nervous system infections category (4.25±0.27) and the lowest score was obtained in the gastrointestinal and hepatobiliary systems category (3.75±0.36). The competency topic-wise distribution of scores is shown in terms of descriptive statistics in Table 2.

**Table 2:** Central patterns of scores in various categories in competency-based medical education curriculum in microbiology MI: microbiology competency category; Q1: first quartile; Q3: third quartile; SD: standard deviation; SEM: standard error of the mean; CI: confidence interval

Parameter	MI 1	MI 2	MI 3	MI 4	MI 5	MI 6	MI 7	MI 8
Minimum	3.5	3.5	3	3.67	3.83	2.33	3.5	3.17
Q1	3.67	3.67	3.54	4.04	4	4	4	4
Median	4.17	4.08	3.83	4.17	4.17	4.17	4.08	4.08
Q3	4.17	4.17	4	4.33	4.5	4.33	4.33	4.33
Maximum	4.33	4.5	4.17	4.33	4.67	4.5	4.67	4.5
Mean	3.96	4	3.75	4.17	4.25	4.01	4.13	4.07
SD	0.33	0.31	0.36	0.2	0.27	0.58	0.3	0.37
SEM	0.094	0.089	0.1	0.058	0.078	0.17	0.087	0.11
Lower 95% CI	3.75	3.8	3.52	4.04	4.08	3.65	3.93	3.83
Upper 95% CI	4.17	4.19	3.98	4.29	4.42	4.38	4.32	4.31

**Figure 4:**
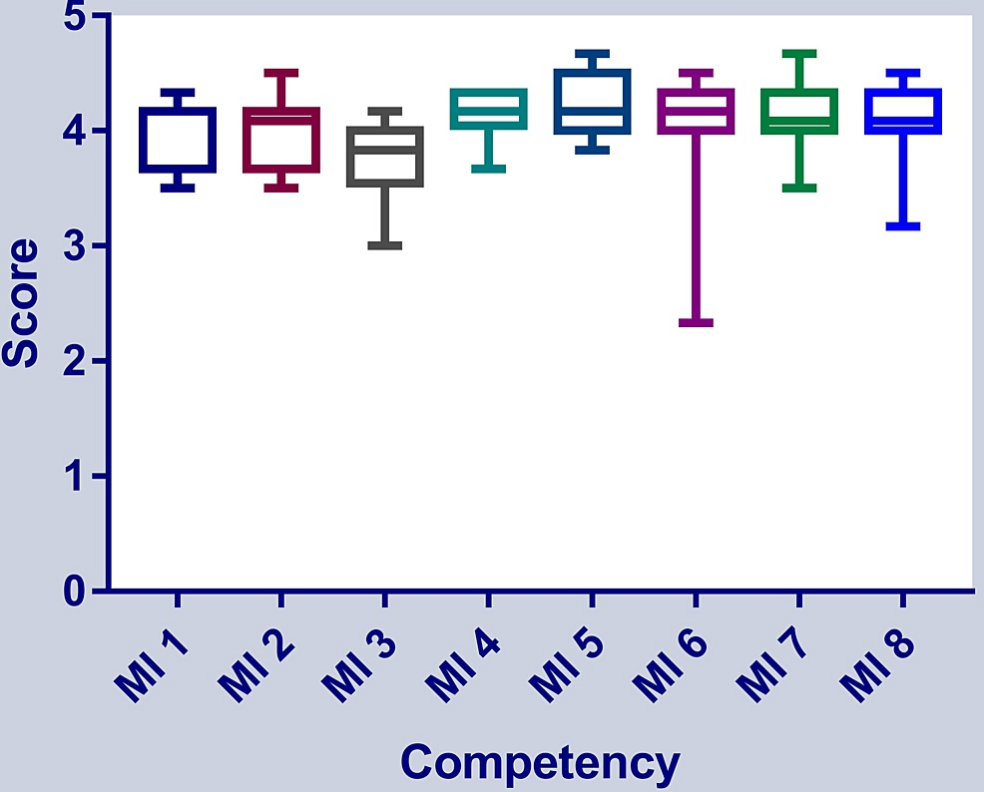
Comparison of scores among various categories in competency-based medical education curriculum in microbiology MI: microbiology competency category

When we compared the scores among various categories according to CBME, we found that there was an overall difference (p=0.02) among the scores, indicating that the application's performance may vary in different topics in microbiology. The comparison is shown in Figure 4.

## Discussion

**Figure 5:**
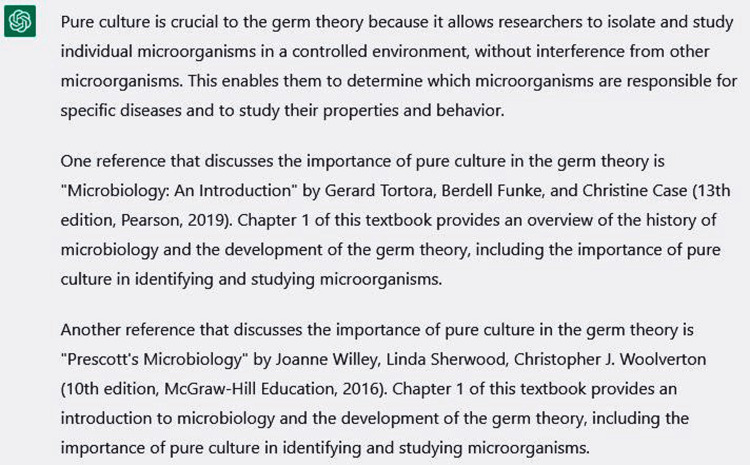
Response of ChatGPT to the question “Answer the following question with two textbook references - Why pure culture is crucial to the germ theory?”

Based on our findings, ChatGPT is capable of providing answers to both first- and second-order knowledge questions with approximately 80% accuracy. This corroborates the findings of Sinha et al [4]. There was no difference in the application's capability to solve both types of questions. Hence, this program can be used by medical students in studying microbiology. In many institutions, self-directed learning (SDL) is practiced where a student collects relevant information from various sources to study a topic. SDL is also an important component of the competency-based curriculum recently introduced in India by the National Medical Commission of India in 2019. This SDL learning approach places medical professionals in charge of their own learning, allowing them to tailor their learning experiences to their individual needs and take ownership of their learning outcomes. By promoting a learner-centered approach, SDL empowers medical professionals to identify their strengths and weaknesses, set goals for improvement, and apply new knowledge and skills to their clinical practice [11]. The CBME curriculum has assigned hours of study for SDL in every subject of medical science. ChatGPT can assist medical students in self-directed learning by providing access to relevant information and answering questions. Medical students can ask questions about specific diseases, treatments, or procedures. The major advantage of the model is personalized learning it provides for the individual needs of medical students. ChatGPT can provide answers with the aid of literature resources to help students understand the concepts and cross-check them. An example is shown in Figure 5.

Across different competency modules, the score was significantly different. The highest score was found for questions related to central nervous system infections and the lowest score was obtained in the gastrointestinal and hepatobiliary systems. The underlying cause may be attributed to the limitation of training the AI system. Any deviation from the uniformity of difficulty may be another reason for the different levels of performance of the language model. Hence, there is scope for further improvement of the model to make it more suitable for academic usage. The applicability of language models like ChatGPT in academic usage is wide-ranging and constantly expanding, and they can be used to enhance various aspects of academic work, including research, writing, teaching, and learning [12,13].

This study has certain limitations. We only evaluated the capability of ChatGPT in answering first- and second-order knowledge questions on the subject of microbiology, and the findings may not be generalizable to other subjects or domains. Secondly, the study only used a single user to converse with ChatGPT. Conversations with another user at different time points may generate a different response. Furthermore, paraphrasing the questions showed variations in the answers. The scoring of the responses was subjective; hence, evaluation bias may be present even after taking the average of three scores from three evaluators.

## Conclusions

The study showed that ChatGPT is a potential tool for answering both first- and second-order knowledge questions with equal vigor in the field of microbiology. The accuracy rate of approximately 80% indicates that ChatGPT has the capability to be a valuable automated question-answering system for students. Although the model showed high accuracy, there is still room for improvement, and it is necessary to fine-tune the model to enhance its performance to achieve nearly 100% accuracy. In the future, with further improvement, ChatGPT-like programs may be used for academic purposes.
